# LncRNA gm40262 promotes liver fibrosis and parasite growth through the gm40262-miR-193b-5p-TLR4/Col1α1 axis

**DOI:** 10.1128/mbio.02287-24

**Published:** 2025-02-25

**Authors:** Tingli Liu, Guiting Pu, Liqun Wang, Ziyu Ye, Hong Li, Rui Li, Yanping Li, Xiaola Guo, William C. Cho, Hong Yin, Yadong Zheng, Xuenong Luo

**Affiliations:** 1 State Key Laboratory for Animal Disease Control and Prevention, Key Laboratory of Veterinary Parasitology of Gansu Province, Lanzhou Veterinary Research Institute, Chinese Academy of Agricultural Sciences (CAAS), Lanzhou, Gansu Province, China; 2 Department of Medical Laboratory, Fenyang College of Shanxi Medical University, Fenyang, China; 3 Key Laboratory of Applied Technology on Green-Eco-Healthy Animal Husbandry of Zhejiang Province, Zhejiang Provincial Engineering Laboratory for Animal Health Inspection and Internet Technology, Zhejiang International Science and Technology Cooperation Base for Veterinary Medicine and Health Management, China-Australia Joint Laboratory for Animal Health Big Data Analytics, College of Animal Science and Technology, College of Veterinary Medicine of Zhejiang A&F University, Hangzhou, Zhejiang province, China; 4 Department of Clinical Oncology, Queen Elizabeth Hospital, Hong Kong, China; 5 Jiangsu Co-Innovation Center for the Prevention and Control of Important Animal Infectious Disease and Zoonoses, Yangzhou University 38043 , Yangzhou, Jiangsu Province, China; University of Michigan-Ann Arbor, Ann Arbor, Michigan, USA

**Keywords:** *E. multilocularis*, liver fibrosis, gm40262, miR-193b-5p, TLR4, Col1α1

## Abstract

Alveolar echinococcosis (AE) is a severe and life-threatening parasitic disease caused by *Echinococcus multilocularis*. Liver fibrosis is a significant pathological feature of advanced AE, characterized by the excessive production and accumulation of extracellular matrix (ECM). However, the precise underlying mechanism remains largely unknown. In this study, we show that the long noncoding RNA gm40262, predominantly expressed in hepatic stellate cells (HSCs), is upregulated in AE. Interestingly, its knockdown leads to liver fibrosis resolution, accompanied by a substantial suppression of parasite growth. Gm40262 functions by targeting miR-193b-5p to activate HSCs and stimulate their proliferation in a TGF-β-dependent manner, thereby promoting ECM production by upregulating Col1α1. Moreover, gm40262 is also involved in inflammation through the gm40262-miR-193b-5p-TLR4 axis. Our findings suggest that gm40262 plays a pivotal role in parasite-induced liver fibrosis through multiple mechanisms, highlighting its potential as a therapeutic target for hepatic fibrosis.

**IMPORTANCE:**

*Echinococcus multilocularis* is a tiny parasite with significant medical implications. The chronic parasitism of *E. multilocularis* in the liver generally leads to liver fibrosis, but the underlying mechanisms are poorly understood. We herein show that gm40262, a long noncoding RNA predominantly expressed in hepatic stellate cells (HSCs), is involved in hepatic fibrogenesis during infection by activating HSCs and promoting extracellular matrix production. The gm40262-orchestrating fibrogenesis occurs through the gm40262-miR-193b-5p-TLR4 and gm40262-miR-193b-5p-Col1α1 axes. The knockdown of gm40262 remarkably alleviates liver fibrosis, with decreased parasite growth. Our findings reveal a key role of gm40262 in liver fibrosis during *E. multilocularis* infection, rendering it a therapeutic target for hepatic fibrosis.

## INTRODUCTION

Alveolar echinococcosis (AE) is a devastating zoonotic parasitic disease caused by *Echinococcus multilocularis* infection. It is recognized as one of the most severe foodborne parasitic diseases, with a global disease burden of approximately 666,434 disability-adjusted life years per annum (1, 2). Clinical diagnosis of AE is often made at an advanced stage, and the primary treatment involves surgical excision combined with drug treatment, such as albendazole and praziquantel, but the prognosis is very poor (3, 4). The liver is the primary organ affected by the parasite, and the continuous leakage of hydatid cyst fluid (HCF) from *E. multilocularis* larvae causes substantial damage and lesions in the liver tissue (5). Consequently, chronic infection leads to pathological changes, including liver structural disorder, liver fibrosis, and liver necrosis (6, 7). Liver fibrosis, particularly at the middle and advanced stages of infection, is highly detrimental and can progress irreversibly to cirrhosis or even liver cancer if left untreated (8, 9). Therefore, it is crucial to identify effective treatment interventions to regress liver fibrosis.

Liver fibrosis is a gradual process characterized by the accumulation of extracellular matrix (ECM), primarily composed of collagen, elastin, proteoglycan, and aminoglycan. Type I collagen consists of two collagen α1 chains (Col1α1) and one collagen α2 chain (Col1α2). Increasing studies have shown that the abnormal expression of Col1α1 is related to many diseases, including fibrosis and cancer (10, 11). In *Schistosoma japonicum*, a fluke causing liver fibrosis through multiple mechanisms (12), miR-29b-3p was found to directly target both Col1α1 and Col3α1, and its overexpression reduced the synthesis of type I and type III collagens, ultimately promoting fibrosis resolution (11). Similarly, the hypoxia-inducible factor-1α subunit has been identified as a potential target in a mouse model of lung fibrosis due to its significant inhibition of Col1α1 expression (13). Hepatic stellate cells (HSCs), a type of non-parenchymal cells accounting for approximately 5% of total liver cells, are a driving factor of fibrosis (14). In normal conditions, quiescent HSCs maintain ECM homeostasis, but they become activated upon liver injury. Therefore, the activation of HSCs is a key feature of liver fibrosis and is characterized by excessive ECM production (15, 16).

Inflammation acts as a bridge between liver injury and fibrosis and is closely associated with fibrosis progression. Prolonged activation of inflammatory responses results in continuous activation of HSCs, causing them to acquire fibroblast characteristics and secrete a large amount of ECM that accumulates in the liver, ultimately leading to fibrosis (17). In this process, Toll-like receptor 4 (TLR4) is a key signaling molecule that links inflammation to fibrosis. Activation of TLR4 signaling in HSCs induces the expression of various inflammatory cytokines, including IL-1β, IL-6, TNF-α, and TGF-β1. Among these, TGF-β1 is a key activator of HSCs, promoting the synthesis of ECM components (10, 18).

Recently, long non-coding RNAs (lncRNAs) have received attention for their beneficial effects on fibrosis in various organs, such as the liver, heart, lungs, and kidneys (19). Several lncRNAs, including MEG3 (20, 21), GAS5 (22, 23), and Gm5091 (24), have been found to inhibit liver fibrosis, suggesting their potential as therapeutic targets for liver fibrosis. Our previous study found that the expression of fibrosis-related genes Col1α1 and α-SMA was significantly increased in HSCs of mice infected with *E. multilocularis*. Moreover, a number of lncRNAs were differentially expressed, including gm40262, which was predicted to be involved in liver fibrosis via multiple signaling pathways although its precise role remained unclear (25). In the present study, we found that gm40262 was upregulated in HSCs after *E. multilocularis* infection. Knockdown of gm40262 inhibited parasite-induced liver fibrosis by alleviating inflammation, HSCs’ activation, and Col1α1 production through multiple mechanisms, highlighting its potential as a promising target for intervention against liver fibrosis caused by *E. multilocularis*.

## MATERIALS AND METHODS

### Cells, parasites, and animal infection

The mouse HSC cell line was purchased from Bluebio Life Sciences Biological Technology (China) and cultured in DMEM medium (Gibco, USA) supplemented with 10% fetal bovine serum (Gibco, USA) at 37°C with 5% CO_2_. The isolation of primary mouse HSCs and parasites, as well as the *in vitro* infection model used in this study, was performed as previously described (25).

Three short hairpin RNAs (shRNAs) for gm40262 were designed and screened *in vitro* (Table S1). For gm40262 knockdown, adeno-associated virus (AAV8)-3in1-shRNA-gm40262-GFP was prepared by Vigene Biosciences (China), and AAV8-U6-shRNA-CMV-GFP was used as a control. Six-week-old BALB/c mice were randomly divided into three groups: phosphate-buffered saline (PBS) group with an injection of PBS (*n* = 9), AAV8-U6 group with an injection of AAV8-U6-shRNA-CMV-GFP (*n* = 9), and AAV8-si-gm40262 group with an injection of AAV8-3in1-shRNA-gm40262-GFP (*n* = 9). A dose of 2 × 10^11^ vg of AAV8-U6-shRNA-CMV-GFP or AAV8-3in1-shRNA-gm40262-GFP in 100 µL PBS was administrated per mouse via the tail vein. After 15 days, 1,000 protoscoleces were intraperitoneally inoculated into each mouse. Mice were humanely slaughtered at different time points as indicated, and samples were collected for later use.

### Histological and immunohistochemical staining analysis

Hematoxylin and eosin (HE) staining was performed to evaluate liver pathological changes. In short, the liver tissues were fixed in a 4% formalin solution and then embedded in paraffin. Subsequently, sections were prepared and stained with HE. Histological fibrosis was detected by Masson’s staining (Wuhan, China). For immunohistochemical analysis, the liver tissues were prepared for frozen sections and incubated with α-SMA (1:200, Abcam) and Col1α1 (1:100, Abcam) antibodies, respectively. The positive areas were quantified using ImageJ.

### Cell transfection

The pcDNA3.1 plasmid (Invitrogen, USA) was used to construct the gm40262 overexpression vector, designated as pcDNA3.1-gm40262. Small interfering RNA (siRNA; Table S2) against gm40262, scramble RNA (si-nc), miR-193b-5p mimics and inhibitors, and negative controls were purchased from RiboBio (China). For the transfection of HSCs and 293T cells, Lipofectamine 3000 (Invitrogen, USA) was used according to the manufacturer’s instructions. The final concentration of pcDNA3.1-gm40262 was 1,500 ng/mL, and all the treatment concentrations of gm40262 siRNA, siRN5AA negative control, miR-193b-5p mimics, and negative control were 100 nM.

### Total RNA extraction and reverse transcription-quantitative polymerase chain reaction

Total RNA extraction and reverse transcription-quantitative polymerase chain reaction (RT-qPCR) were performed as previously described (25). In short, using the HiScript III First-strand cDNA Synthesis Kit (Vazyme, Germany), 1 µg RNA was reversely transcribed to cDNA. Subsequently, RT-qPCR was carried out using the ABI 7500 system (Thermo Fisher Scientific, USA), with the reaction conditions as follows: 95°C for 10 min and 40 cycles of 95°C for 15 s and 60°C for 1 min. The relative expression levels of genes were calculated using the formula of 2^−ΔΔ Ct^. The primers used in this study are listed in Table S3.

### Western blotting

Liver tissues and cells were lysed in RIPA buffer for 30 min, followed by centrifugation at 12,500  × *g* for 20 min at 4°C to remove insoluble components. Protein concentrations were measured using the BCA Protein Assay Kit (Beyotime, China). A total of 30 µg proteins were separated by 12% SDS-PAGE and then transferred onto PVDF membranes. After being incubated with 5% BSA for 1 h, the membranes were incubated with antibodies against GAPDH (1:3,000, Abcam), TLR4 (1:1,000, Beyotime), IL-1β (1:2,000, Santa Cruz), TGF-β (1:1,000, Beyotime), p-Smad2 (1:1,000, Beyotime), p-Smad3 (1:1,000, Beyotime), Collα1 (1:1,000, Abcam), and α-SMA (1:1,000, Abcam) at 4°C overnight, respectively. The corresponding HRP-conjugated secondary antibodies (goat anti-mouse IgG for GAPDH, IL-1β, and α-SMA, and goat anti-rabbit IgG for TLR4, p-Smad2, p-Smad3, and Collα1, 1:3,000, Biodragon) were subsequently incubated. After exposure using chromogenic reagents A and B (Beyotime, China), the blots were quantified using ImageJ.

### Dual luciferase reporter assay

The fragments of wild-type gm40262 (gm40262-WT), TLR4 (TLR4-WT), and Col1α1 (Col1α1-WT) and mutated gm40262 (gm40262-MUT), TLR4 (TLR4-MUT), and Col1α1 (Col1α1-MUT) were inserted into the pmirGLO vector (Promega, USA), respectively. Every construct was co-transfected into HEK293T cells together with miR-193b-5p mimics or negative control. After 48 h, luciferase activity was determined by Promega Glomax 96 (Promega, USA).

### Cell proliferation assay and cell cycle analysis

In 96-well plates, 3 × 10^3^ HSCs were seeded per well. When the cell confluence reached 50%, HCF previously prepared in our lab was added with a final concentration of 0.8 mg/mL and incubated for 24 h. Then, 10 µL of Cell Counting Kit-8 solution (Dojindo, Japan) was added into each well. After further cultivation for 4 h, OD values at 450 nm were measured using the Epoch Microplate Spectrophotometer (BioTek, USA).

The cell cycle was analyzed using the DNA Content Quantitation Assay (Solarbio, China). In brief, HSCs were fixed with 500 µL of 70% precooled ethanol for 2 h and then rinsed with PBS. HSCs were resuspended with 100 µL of RNase A solution and incubated at 37℃ for 30 min. Subsequently, 400 µL of propidium iodide staining solution was added and incubated in the dark at 4°C for 30 min. Finally, the fluorescence intensity at 488 nm was recorded using CytoFLEX (Beckman, China).

### Fluorescence *in situ* hybridization

Three different probes for gm40262 fluorescence *in situ* hybridization (FISH), P1, P2, and P3 (Table S4), were designed and synthesized by Servicebio (Wuhan, China). The FISH assay was performed using liver tissue sections according to the specifications of the manufacturer. Briefly, after dewaxing, repair, and digestion, the paraffin sections were pre-hybridized at 37°C for 1 h, followed by incubation in hybridization solution with the P1 probe at a concentration of 500 nM at 37°C overnight. After washing, the sections were incubated with the P2 probe at 40°C for 45 min. Subsequently, after washing, the sections were incubated with the P3 probe at 40°C for 45 min. The nucleus was stained with DAPI (Sigma-Aldrich, USA), and the cytoplasm was stained with ActinGreen 488 ReadyProbes (Invitrogen, USA). The sections were observed using a fluorescence microscope (Nikon, Japan).

### Statistical analysis

GraphPad Prism 6 was used to perform statistical analyses, and the results were presented as mean  ±  standard deviation. Unpaired Student’s *t*-test and ANOVA were performed to compare the difference in two groups and three or more groups, respectively, with *P* < 0.05 considered statistically significant.

## RESULTS

### *E. multilocularis* infection induces liver fibrosis

To investigate *E. multilocularis*-induced hepatic fibrosis, we analyzed the fibrotic tissues and the expression of related proteins. In comparison with the control group, infected mice exhibited severe hepatocellular necrosis, along with a significant increase in inflammatory cell infiltration (Fig. S1A) and the accumulation of blue-stained collagen fibers 2 and 3 months post-infection (mpi) (*P* < 0.001, Fig. S1B). Furthermore, the expression of α-SMA (Fig. S1C) and Col1α1 (Fig. S1D) in the liver was also significantly elevated. These findings confirm that liver fibrosis develops following *E. multilocularis* infection.

### Gm40262 is upregulated in liver HSCs following *E. multilocularis* infection

**Fig 1:**
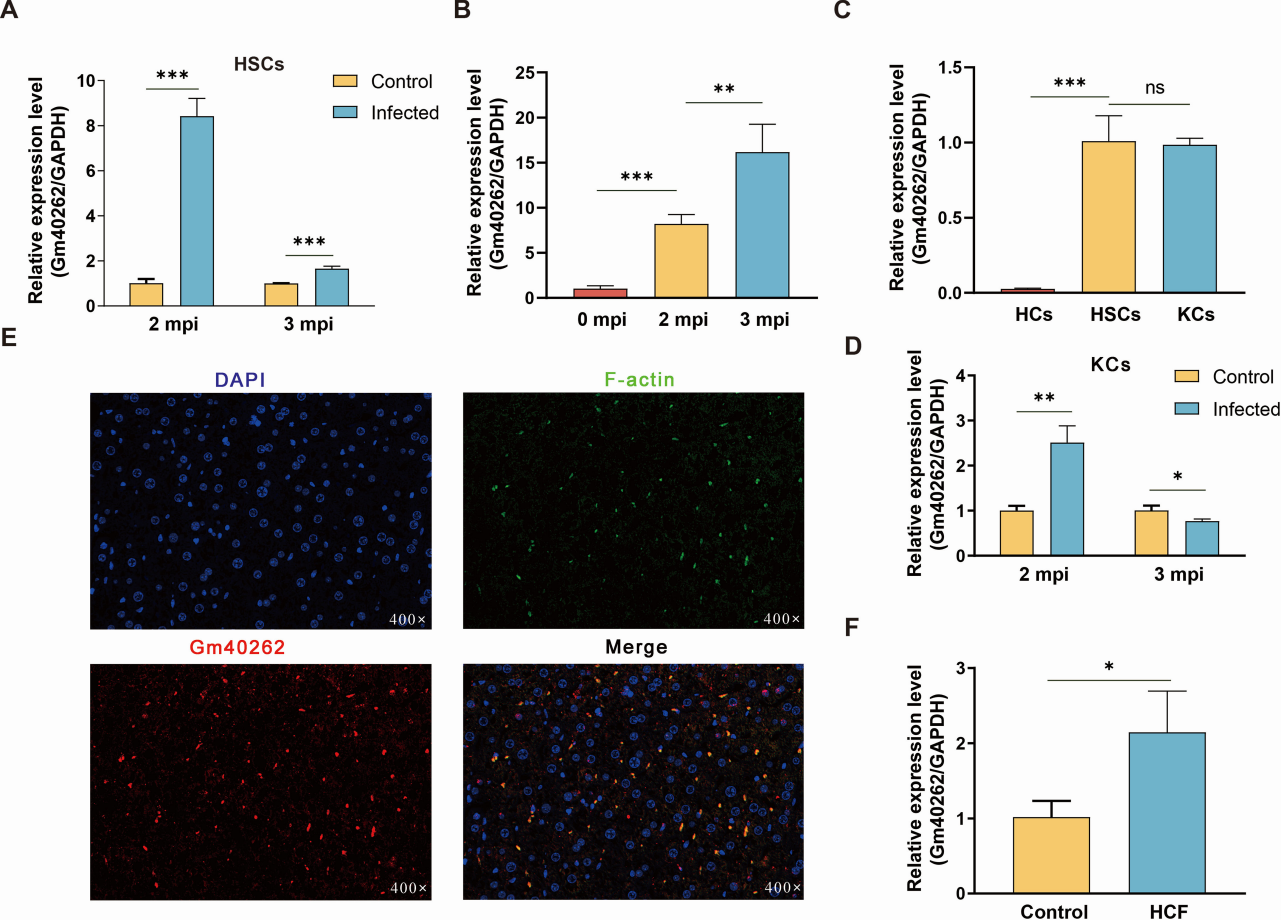
The effects of *E. multilocularis* infection and HCF on the expression of gm40262 (**A and B**) The relative expression level of gm40262 in HSCs in the liver of mice infected with *E. multilocularis* at different times post-infection. (**C**) The relative expression level of gm40262 in three types of cells in the liver of healthy mice by RT-qPCR. (**D**) The relative expression level of gm40262 in KCs at different times after infection by RT-qPCR. (**E**) The location of gm40262 in the liver by FISH assay. (**F**) The relative expression level of gm40262 in HSC cells treated with 0.8 mg/mL HCF. HCs, hepatocytes. *P < 0.05; **P < 0.01; ***P < 0.001; and ns, not significant.

Previously, we reported that gm40262 was one of the significantly upregulated lncRNAs in the HSCs of mice infected with *E. multilocularis* (25). Subsequently, gm40262 was further verified to be significantly upregulated in activated HSCs during infection through RT-qPCR analysis (Fig. 1A and B). Moreover, it was observed to be highly expressed in both HSCs and Kupffer cells (KCs) compared to hepatocytes in healthy mice (Fig. 1C). Additionally, gm40262 exhibited continuous upregulation in activated HSCs but not in KCs post-infection (Fig. 1A and D). Consistently, FISH results indicated that gm40262 was predominantly expressed in F-actin-enriched cells in the liver (Fig. 1E), such as activated HSCs (26). To determine whether upregulation of gm40262 is induced by parasite-origin products, we monitored its expression changes in quiescent HSCs treated with HCF (0.8 mg/mL), exosomes (25 µg/mL), and excretory-secretory products (25 µg/mL) of *E. multilocularis*, all of which were previously prepared in our laboratory (27), respectively. Surprisingly, gm40262 showed significant upregulation only following HCF treatment (Fig. 1F). Knockdown of gm40262 resulted in decreased expression of both α-SMA and Col1α1 in HSCs, even when treated with HCF (Fig. S2A and B), which was previously shown to activate HSCs (28). These results suggest that gm40262 is upregulated in activated HSCs during infection, potentially induced by HCF.

### Knockdown of gm40262 ameliorates liver fibrosis and suppresses parasite growth

**Fig 2:**
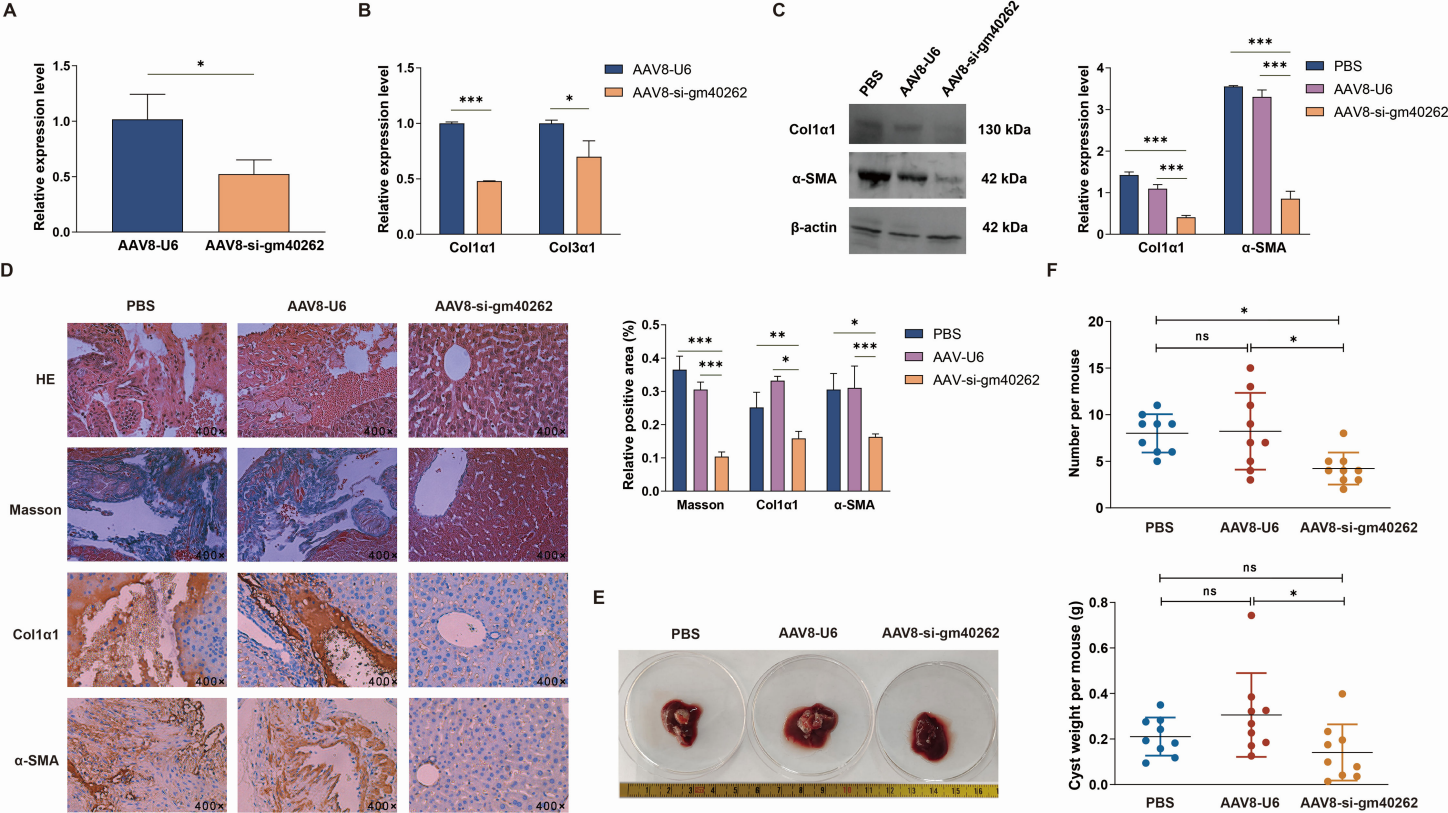
Inhibition of liver fibrosis by gm40262 knockdown. (**A**) The expression of gm40262 in the liver of treated mice by RT-qPCR. (**B**) The expression of fibrosis-related genes in the liver of treated mice by qRT-PCR. (**C**) The expression of fibrosis-related proteins in the liver of treated mice by Western blotting. (**D**) The liver tissues stained by HE, Masson, and α-SMA and Col1α1 immunohistochemistry. (**E**) Cysts in the liver of mice 3 months post-infection. (**F**) The number and weight of cysts in the liver of mice in three groups. *P < 0.05; **P < 0.01; and ***P < 0.001.

The upregulation of gm40262 in activated HSCs suggests a potential role in parasite-induced hepatic fibrosis. To investigate this hypothesis, AAV8-si-gm40262 expressing anti-gm40262 shRNA and AAV8-U6 expressing control shRNA were separately administered to mice, followed by a comparative assessment of liver fibrosis. Fifteen days post-injection, GFP was detected in both the AAV8-U6 and AAV8-si-gm40262 groups but not in the PBS group, indicating the effective expression of the inserted fragments in the liver (Fig. S3A). Correspondingly, the level of gm40262 was significantly reduced in the liver of mice inoculated with AAV8-si-gm40262 compared to those inoculated with AAB8-U6 (*P* < 0.001, Fig. 2A). The decreased expression of gm40262 led to a marked suppression of Col1α1, Col3α1, and α-SMA (Fig. 2B and C). As anticipated, the positive areas of all the collagen, α-SMA, and Col1α1 were considerably lower in the AAV8-si-gm40262 group than in the other groups 3 mpi (*P* < 0.001, Fig. 2D).

To evaluate the impact of liver fibrosis resolution on parasitism, we examined the growth of cysts in the liver of mice. The infection rate in all three groups was 100% (9/9), yet the mean number and total weight of cysts per mouse were significantly lower in the AAV8-si-gm40262 group than in the other groups (*P* < 0.05), with no significant difference between the AAV8-U6 group and the PBS group (Fig. 2E and F). These findings demonstrate that low expression of gm40262 accelerates the regression of liver fibrosis and restricts parasite growth.

### Gm40262 promotes liver fibrosis through the gm40262/miR-193b-5p/Col1α1 axis

**Fig 3:**
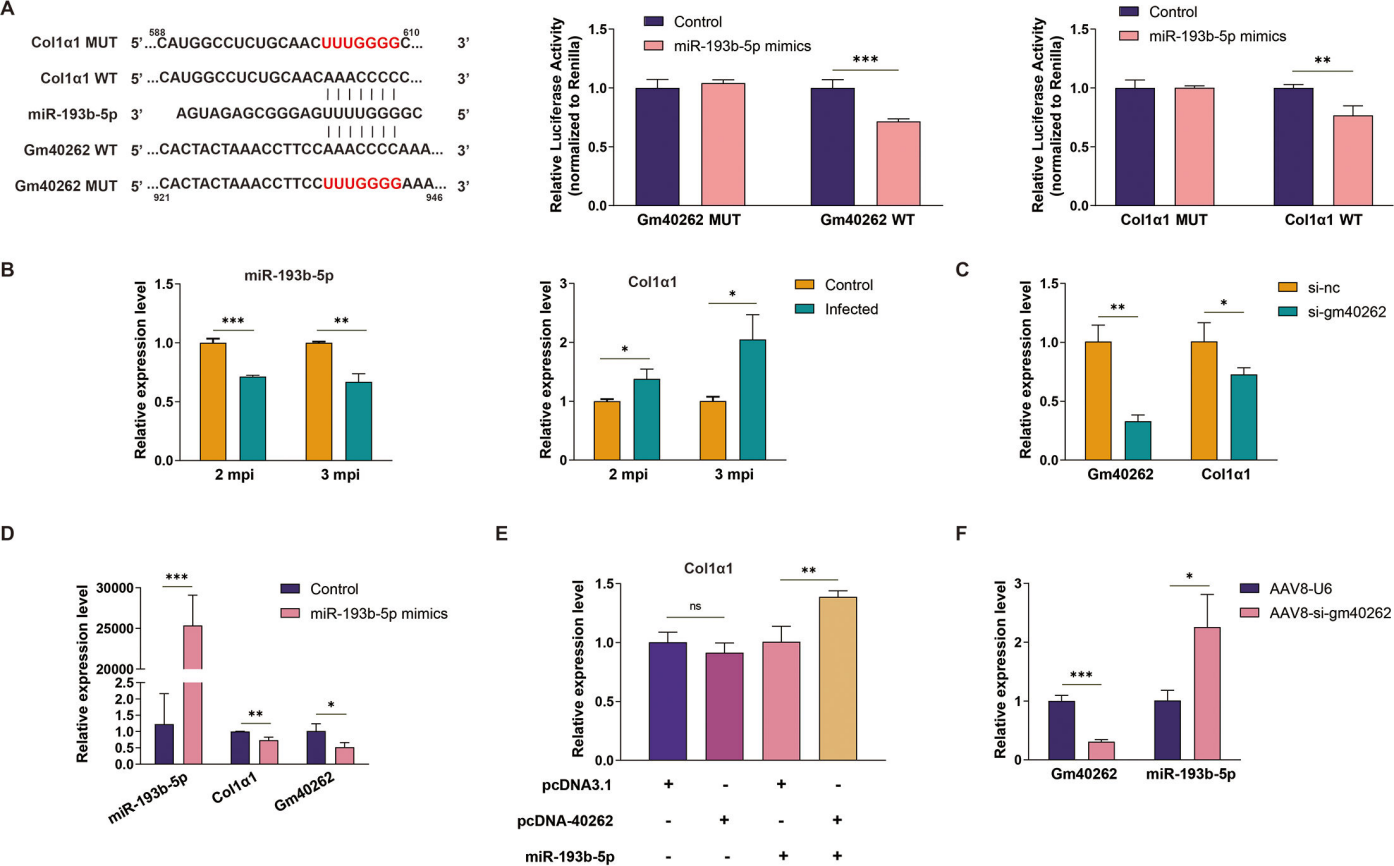
Regulation of Col1α1 by gm40262 through the gm40262/miR-193b-5p/Col1α1 axis. (**A**) Prediction of binding sites (highlighted in red) of gm40262, miR-193b-5p, and Col1α1 using miRanda (https://www.bioinformatics.com.cn/local_miranda_miRNA_target_prediction_120) and RNAhybrid (https://bibiserv.cebitec.uni-bielefeld.de/rnahybrid/) and normalized relative luciferase activity in 293T cells co-transfected with the wild-type (WT) or mutated (MUT) construct and miR-193b-5p mimics and control, respectively. The relative nucleotide positions are indicated by the numbers above and below the sequences. (**B**) The relative expression level of miR-193b-5p and Col1α1 in HSCs in the liver of mice infected with *E. multilocularis* at different times post-infection. (**C**) Effects of gm40262 knockdown on Col1α1 expression in HSCs. (**D**) Effects of increased miR-193b-5p on Col1α1 expression in HSCs. (**E**) Effects of increased gm40262 on Col1α1 expression in HSCs transfected with or without miR-193b-5p mimics. (**F**) The expression of miR-193b-5p after knockdown of gm40262 using AAV8-si-gm40262 *in vivo*. si-nc, siRNA control. *P < 0.05; **P < 0.01; ***P < 0.001; and ns, not significant.

Mounting evidence suggests that lncRNAs regulate target mRNA expression by functioning as competing endogenous RNAs (ceRNAs) (29). Therefore, we hypothesize that gm40262 acts as a miRNA sponge to participate in hepatic fibrosis. It was predicted that gm40262 could act as a sponge of miR-193b-5p and thereby regulate the expression of Col1α1 (Fig. 3A; Fig. S4A). The dual luciferase reporter assay demonstrated that, compared to the control, the fluorescence intensity significantly decreased in cells co-transfected with miR-193b-5p mimics and the gm40262 construct but not with miR-193b-5p mimics and the mutated gm40262 constructs, indicating that miR-193b-5p binds to gm40262 (Fig. 3A). Similarly, miR-193b-5p was confirmed to bind to Col1α1 but not to the mutated Col1α1 construct (Fig. 3A). As expected, miR-193b-5p was downregulated, while Col1α1 was upregulated in the liver of mice 2 and 3 mpi (Fig. 3B). In line with these findings, overexpression of miR-193b-5p led to decreased expression of Col1α1 and Col3α1 (Fig. S5A), while its low expression resulted in elevated expression (Fig. S5B), suggesting a role in ECM production.

To further validate the gm40262/miR-193b-5p/Col1α1 axis, we examined their interdependent expression. Under conditions of gm40262 knockdown and miR-193b-5p overexpression, Col1α1 was significantly downregulated in treated HSCs (Fig. 3C and D). Conversely, the expression level of Col1α1 was significantly restored in HSCs overexpressing miR-193b-5p upon gm40262 overexpression (Fig. 3E). Consistent with the above findings, the expression of Col1α1 was significantly reduced (Fig. 2B and C), while miR-193b-5p was significantly increased in the AAV8-si-gm40262 group compared to the control (*P* < 0.05, Fig. 3F). These results suggest that gm40262 accelerates hepatic fibrosis by inhibiting miR-193b-5p and further promoting Col1α1 expression.

### Gm40262 modulates liver inflammation through the gm40262/miR-193b-5p/TLR4 axis

**Fig 4:**
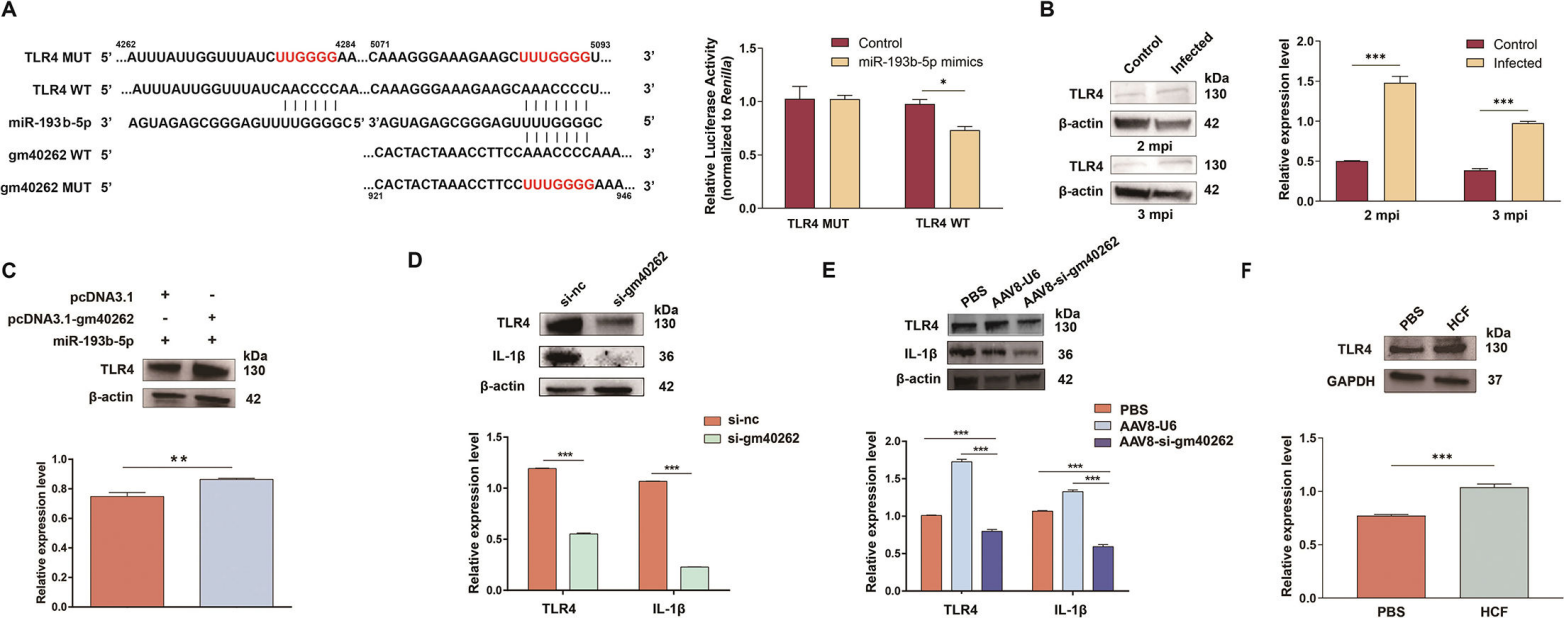
Regulation of TLR4 by gm40262. (**A**) Prediction of binding sites (highlighted in red) of gm40262, miR-193b-5p, and TLR4 using TargetScan (https://www.targetscan.org/vert_71/) and miRPathDBv2.0 (https://mpd.bioinf.uni-sb.de/overview.html) and normalized relative luciferase activity in 293T cells co-transfected with the wild-type (WT) or mutated (MUT) construct and miR-193b-5p mimics and control, respectively. The relative nucleotide positions are indicated by the numbers above and below the sequences. (**B**) The relative expression level of TLR4 in HSCs in the liver of mice infected with *E. multilocularis* at different times post-infection. (**C**) The expression level of TLR4 in miR-193b-5p mimics-transfected HSCs after overexpressing gm40262. (**D**) The expression levels of TLR4 and IL-1β in HSCs after knockdown gm40262. (**E**) The expression levels of TLR4 and IL-1β in liver after knockdown of gm40262 using AAV8-si-gm40262 *in vivo.* (**F**) The relative expression level of TLR4 in HSCs treated with 0.8 mg/mL HCF. si-nc, siRNA control. *P < 0.05 and ***P < 0.001.

miR-193b-5p was also predicted to interact with several inflammatory genes, including TLR4, which has two potential binding sites in its 3′ UTR (Fig. 4A; Table S5). Given the association between liver fibrosis and inflammation, we aimed to investigate whether gm40262 regulates inflammation in the liver during the infection. Indeed, miR-193b-5p was shown to bind to TLR4 but not to the mutated TLR4 construct (Fig. 4A). As expected, TLR4 tended to be upregulated in the livers of infected mice 2 and 3 mpi (Fig. 4B). In HSCs, TLR4 was upregulated after gm40262 overexpression (Fig. 4C), and vice versa (Fig. 4D). Similarly, the expression of TLR4 was significantly decreased in the AAV8-si-gm40262 group compared to the control (*P* < 0.05, Fig. 4E). Due to the upregulation of gm40262 by HCF, we then tested its effects on TLR4 expression. The results showed that HCF did elevate TLR4 expression (Fig. 4F). These results demonstrate that gm40262 induces TLR4 expression by suppressing miR-193b-5p.

**Fig 5:**
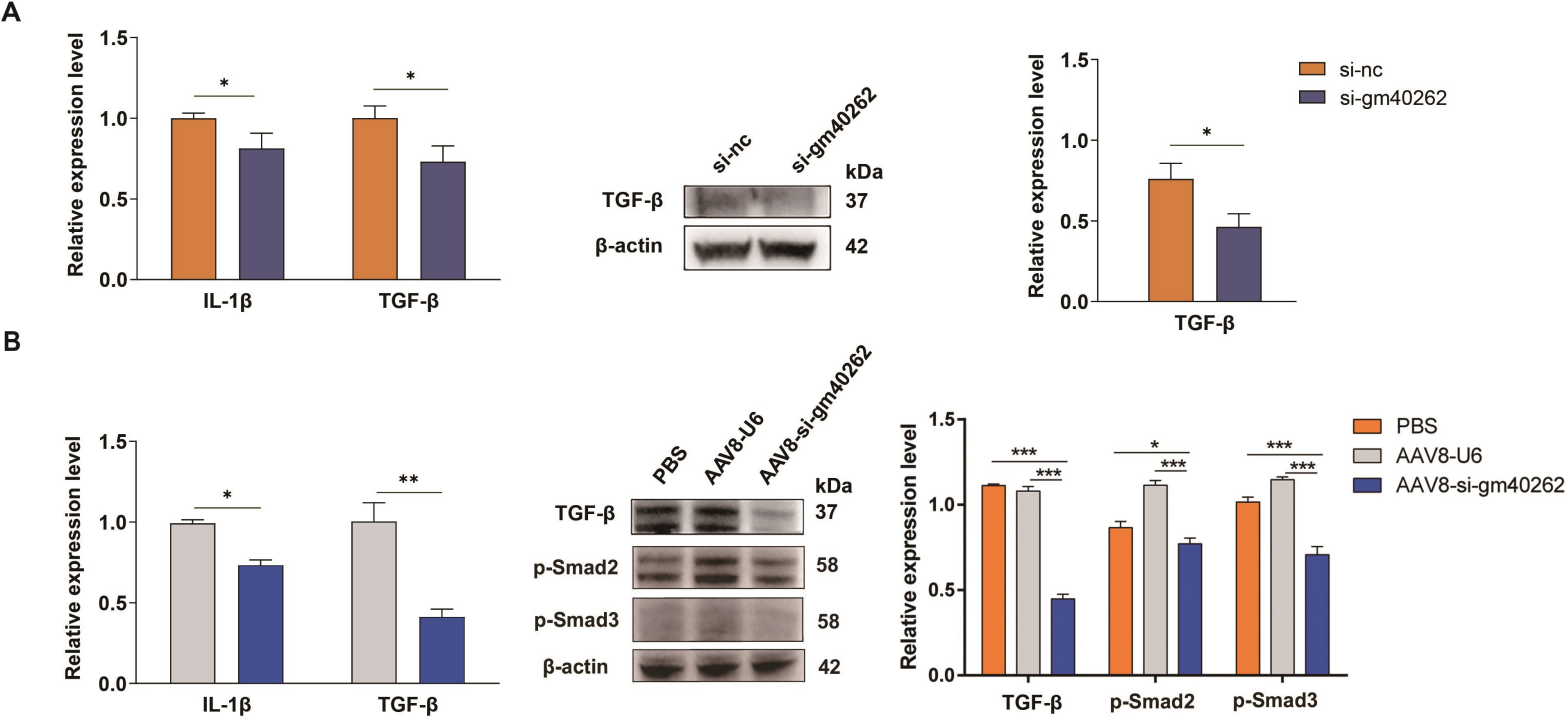
Amelioration of inflammation by gm40262 knockdown. (**A**) The relative levels of IL-1β and TGF-β in HSCs after gm40262 knockdown by RT-qPCR (left panel) or/and Western blotting (right panel). (**B**) The relative levels of IL-1β and TGF-β, p-Smad2, and p-Smad3 in the liver after gm40262 knockdown using AAV8-si-gm40262 by RT-qPCR (left panel) or/and Western blotting (right panel). si-nc, siRNA control. *P < 0.05; **P < 0.01; and ***P < 0.001.

It has been shown that the activation of TLR4 increases the expression of both IL-1β and TGF-β (18, 30), which are activators of quiescent HSCs. We then determined the effects of gm40262 on the expression of these cytokines. In HSCs, both IL-1β and TGF-β decreased after gm40262 knockdown (Fig. 4D and 5A). Moreover, the expression of IL-1β and TGF-β was significantly inhibited in the AAV8-si-gm40262 group compared to the other groups (Fig. 4E and 5B). These results suggest that gm40262 promotes inflammation in the liver by upregulating IL-1β and TGF-β via targeting TLR4.

### Gm40262 promotes HSC proliferation and activation via the TGF-β/Smad signaling pathway

**Fig 6:**
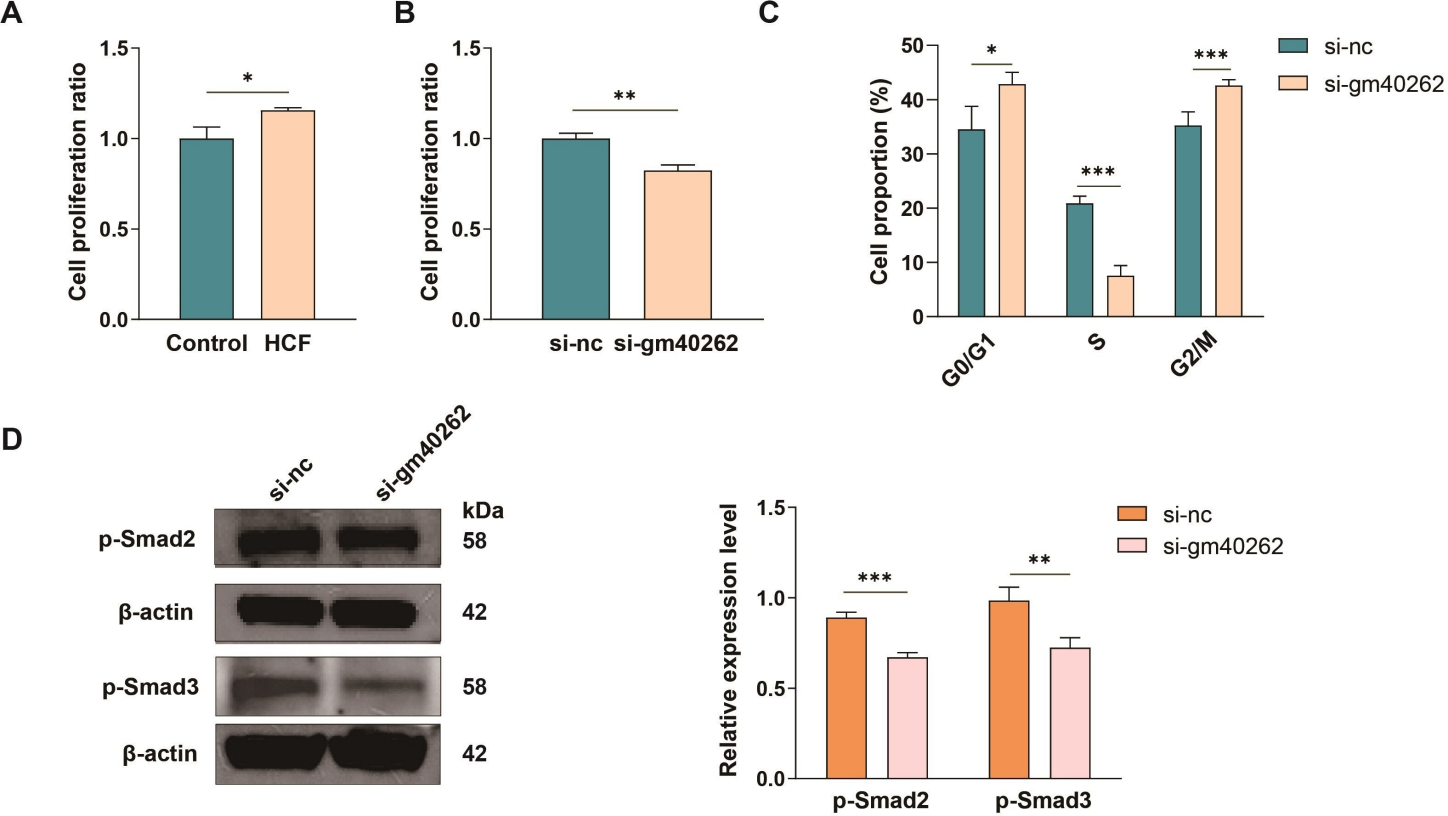
Regulation of HSC activation by gm40262 knockdown via inhibiting the TGF-β/Smad signaling pathway. (**A**) HSC proliferation after treatment with 0.8 mg/mL HCF. (**B**) HSC proliferation after knockdown of gm40262. (**C**) Different cell subpopulations of HSCs in response to gm40262 knockdown by flow cytometry. (**D**) The protein expression levels of p-Smad2 and p-Smad3 in HSCs after knockdown of gm40262. si-nc, siRNA control. *P < 0.05; **P < 0.01; and ***P < 0.001.

As *E. multilocularis* infection induces HSC proliferation (6), we evaluated the effects of gm40262 on HSC proliferation. The results showed that HCF induced HSC proliferation (*P* < 0.05, Fig. 6A) as previously reported (28), but both gm40262 knockdown and miR-193b-5p overexpression reversed this increase (Fig. 6B; Fig. S5C). The decreased HSC proliferation was likely attributed to inhibiting DNA synthesis (*P* < 0.001, Fig. 6C).

TGF-β, a robust stimulator that drives HSC activation via the TGF-β/Smad signaling pathway (31, 32), was inhibited after gm40262 knockdown, suggesting that gm40262 activates HSCs by upregulating TGF-β. Consistently, the expression of p-Smad2 and p-Smad3 was suppressed after gm40262 knockdown (Fig. 6D). In infected mice administered with AAV8-si-gm40262, both p-Smad2 and p-Smad3 declined compared to the control (Fig. 5B). These results suggest that gm40262 is involved in HSC activation through the TGF-β/Smad signaling pathway.

## DISCUSSION

*E. multilocularis* larvae primarily parasitize the liver and lung of intermediate hosts, with the liver being the most affected organ (33). Due to constant leakage of HCF, *E. multilocularis* larval infection causes local liver fibrosis and tissue necrosis (34, 35). Recent studies have shown that lncRNAs play a role in regulating the pathological process of liver fibrosis. Some lncRNAs, such as lnc-SCARNA10, linc-SCRG1, and lnc-LFAR1 (36–38), are profibrogenic, while others like MEG3, GAS5, and GM5091 (20, 23, 24) are antifibrogenic. Increasing evidence has demonstrated the existence of lncRNA-mediated competitive RNA crosstalk in liver fibrosis (39, 40). This study elucidated the role of both the gm40262/miR-193b-5p/TLR4 axis and the gm40262/miR-193b-5p/Col1α1 axis in liver fibrosis during *E. multilocularis* infection. The inhibition of gm40262 led to the resolution of liver fibrosis by upregulating miR-193b-5p, thereby downregulating TLR4 and Col1α1.

HSCs, responsible for collagen production, are key players in the initiation and progression of liver fibrosis. HSC activation is a critical event in the onset of liver fibrosis (10). Under normal conditions, HSCs are quiescent but transform into activated HSCs upon liver injury, producing α-SMA and ECM components, such as Col1α1, Col1α3, and Col1α4 (41). The activation of the TLR4/NF-κB signaling pathway sustains continuous HSC activation, leading to collagen secretion and accelerating liver fibrosis (42, 43). This study found that *E. multiloculari*s infection promoted the expression of gm40262 and Col1α1 in HSCs while downregulating miR-193b-5p. Further evidence indicates that gm40262 acts as a ceRNA by sequestering miR-193b-5p and regulating TLR4 expression, which in turn influences cytokine expression involved in inflammatory responses in the liver.

Various pathways, such as Notch, TGF-β-Smad, PI3K/AKT, NF-κB, and MAPK pathways, are involved in HSC activation and proliferation in liver fibrosis (37, 44–46). In this study, TGF-β was significantly increased in the HSCs highly expressing gm40262 but decreased in the HSCs with low expression of gm40262, indicating that gm40262 regulates the TGF-β-Smad pathway. Consistent with this finding, the knockdown of gm40262 inhibited the phosphorylation of both Smad2 and Smad3 in HSCs, leading to the inhibition of the TGF-β/Smad signaling pathway and subsequent downregulation of Col1α1 and α-SMA. Therefore, the knockdown of gm40262 reduces the expression of Col1α1 and α-SMA through the TGF-β/Smad signaling pathway.

To assess whether gm40262 knockdown affects liver fibrosis regression, gene therapy targeting gm40262 was utilized in BALB/c mice. The choice of an appropriate AAV vector depends on various factors, including different serotypes, tissue tropism, and immunogenicity risks. For liver-targeted gene therapy, several carrier serotypes are available, such as AAV2, AAV5, AAV8, AAV9, and AAV10 (47). Among these, AAV8 is favored due to its liver tropism (48, 49). Thus, this study employed AAV8 to deliver the si-gm40262 to the liver. Consistent with the *in vitro* findings, gm40262 knockdown resulted in the repression of TLR4, IL-1β, Col1α1, and α-SMA, along with an increase in miR-193b-5p expression. Moreover, the levels of fibrosis-related proteins and collagens decreased, leading to the alleviation of liver fibrosis. It was observed that the growth and development of parasites were significantly hindered by low gm40262 expression. Further investigation into the precise mechanism by which gm40262 promotes *E. multilocularis* growth and development is warranted.

Kupffer cells have also been identified as another key regulator of liver fibrosis (50, 51). In this study, it was found that gm40262 was enriched and differentially expressed in Kupffer cells during *E. multilocularis* infection, suggesting a role in their biological functions. The potential synergistic role of gm40262 in Kupffer cells in hepatic fibrosis warrants further exploration.

In conclusion, this study has elucidated the mechanisms of the gm40262/miR-193b-5p/TLR4/Col1α1 axis HSC activation and demonstrated that repression of gm40262 slows down the development of liver fibrosis induced by *E. multilocularis* infection. These findings suggest that gm40262 represents a promising novel therapeutic target for the treatment of liver fibrosis.

## SUPPLEMENTAL MATERIAL

Supplemental material mbio.02287-24-s0001.docx Fig. S1-S5; Tables S1 to S5.

ASM does not own the copyrights to Supplemental Material that may be linked to, or accessed through, an article. The authors have granted ASM a non-exclusive, world-wide license to publish the Supplemental Material files. Please contact the corresponding author directly for reuse.
