# Supplementary material for: LncRNA gm40262 promotes liver fibrosis and parasite growth through the gm40262-miR-193b-5p-TLR4/Col1α1 axis
Source: mBio. 2025 Feb 25;16(4):e02287-24. doi: 10.1128/mbio.02287-24 (PMC11980551; doi:10.1128/mbio.02287-24)
Supplement: Supplemental material — Fig. S1-S5; Tables S1 to S5. [file mbio.02287-24-s0001.docx]

**Supplementary materials**


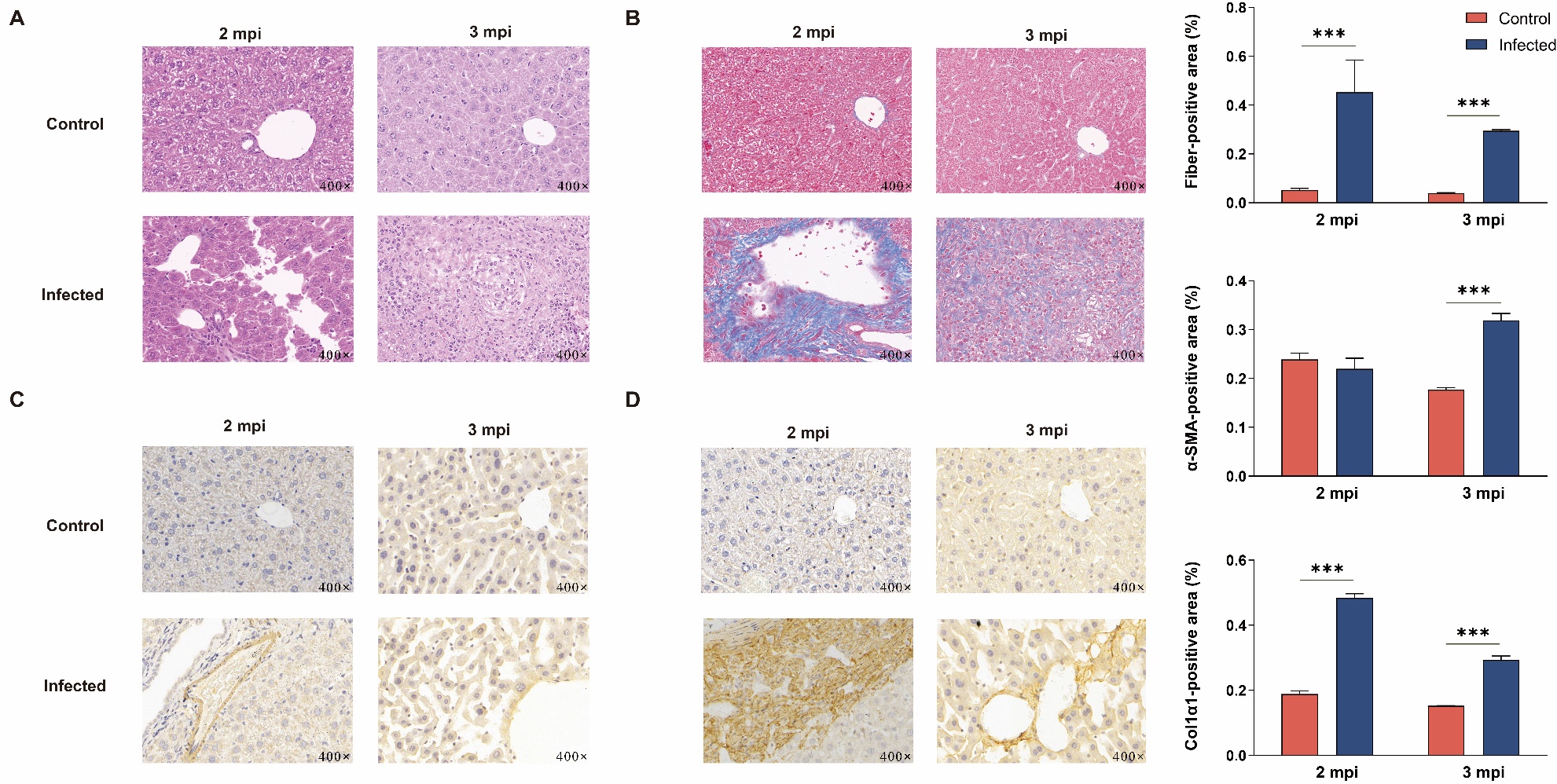


**Fig. S1 Liver fibrosis in the BALB/c mice infected with *Echinococcus multilocularis*** The liver tissues were stained by hematoxylin-eosin (HE, **A**), Masson **(B)**, and immunohistochemistry **(C-D)**, respectively. In short, the liver tissues of mice 2 and 3 months post infection (mpi) were fixed in 4% formalin solution and embedded in paraffin, followed by staining with HE and Masson staining reagents, respectively. The frozen sections were also prepared and treated by α-SMA (1:200, **C**) and Col1α1 (1:100, **D**) antibodies, respectively. The positive areas were quantified using ImageJ. ‘***’，*P* < 0.001.


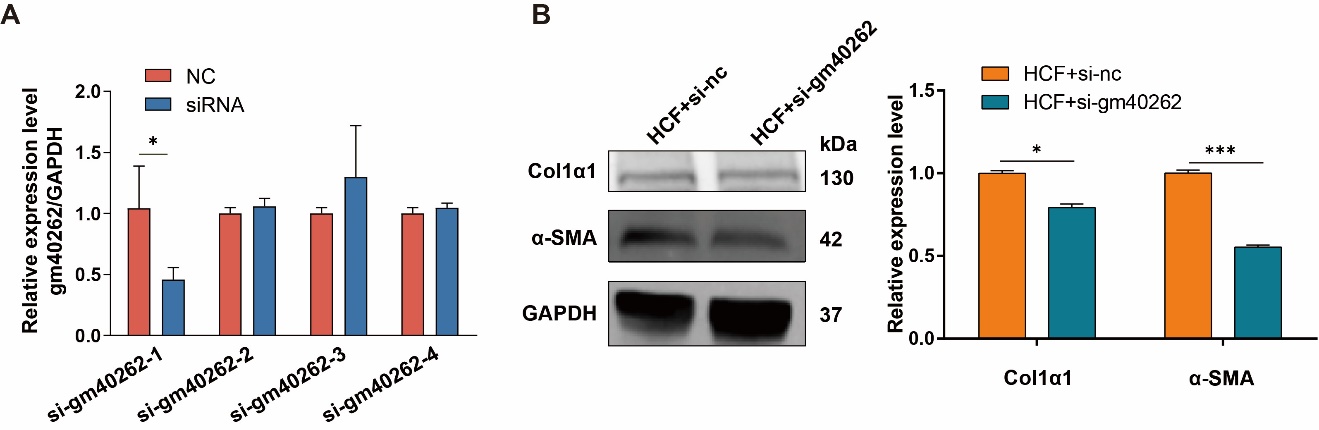


**Fig. S2 The effect of gm40262 knockdown on hydatid cyst fluid (HCF)-activated HSCs** Four different siRNAs against gm40262, si-gm40262-1-4, were designed and then the levels of gm40262 were detected by qPCR after 24 h transfection **(A)**. HSCs were first treated with 0.8 mg/mL HCF and then the levels of α-SMA and Col1α1 were comparatively assessed in treated HSCs after 24 h transfection with siRNA-gm40262 and siRNA control (si-nc), respectively **(B)**. ‘NC’, negative control; ‘*’, *P* < 0.05; ‘***’, *P* < 0.001.


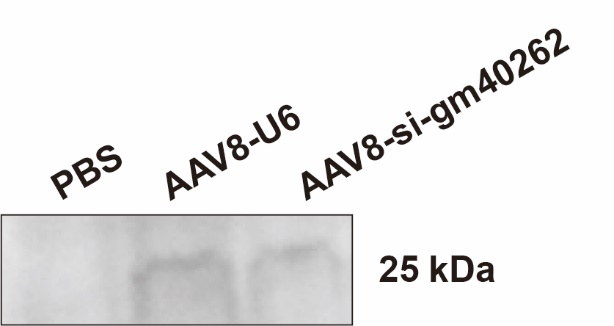


**Fig. S3 Effective expression of gm40262 in the liver of mice inoculated with AAV8-si-gm40262** In short, 6 week-old BALB/c mice were injected with 100 µl PBS, 2×10^11^ vg of AAV8-U6-shRNA-CMV-GFP and AAV8-3in1-shRNA-gm40262-GFP via the tail vein, respectively. After 15 d, 1,000 of protoscoleces were intraperitoneally inoculated into each of mice. Mice were humanely slaughtered 3 months post infection. The level of GFP (1:5000, Abmart) in the liver of one mouse in each group were detected randomly by Western blotting.


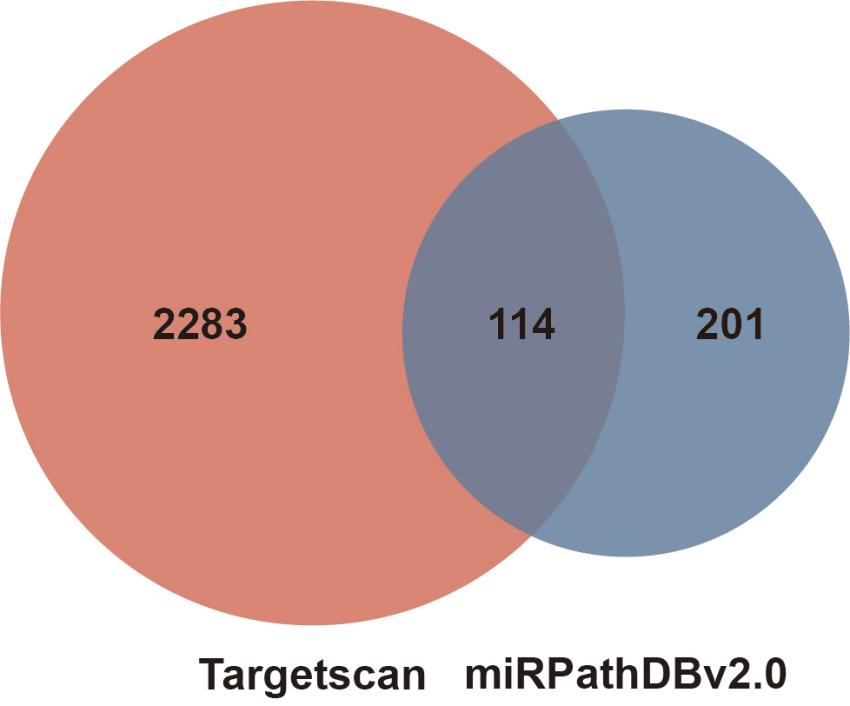


**Fig. S4 Venn diagram displaying the number of target genes of miR-193b-5p predicted by**

**TargetScan (https://www.targetscan.org/vert_71/) and miRPathDBv2.0 (https://mpd.bioinf.uni-sb.de/overview.html)**

**
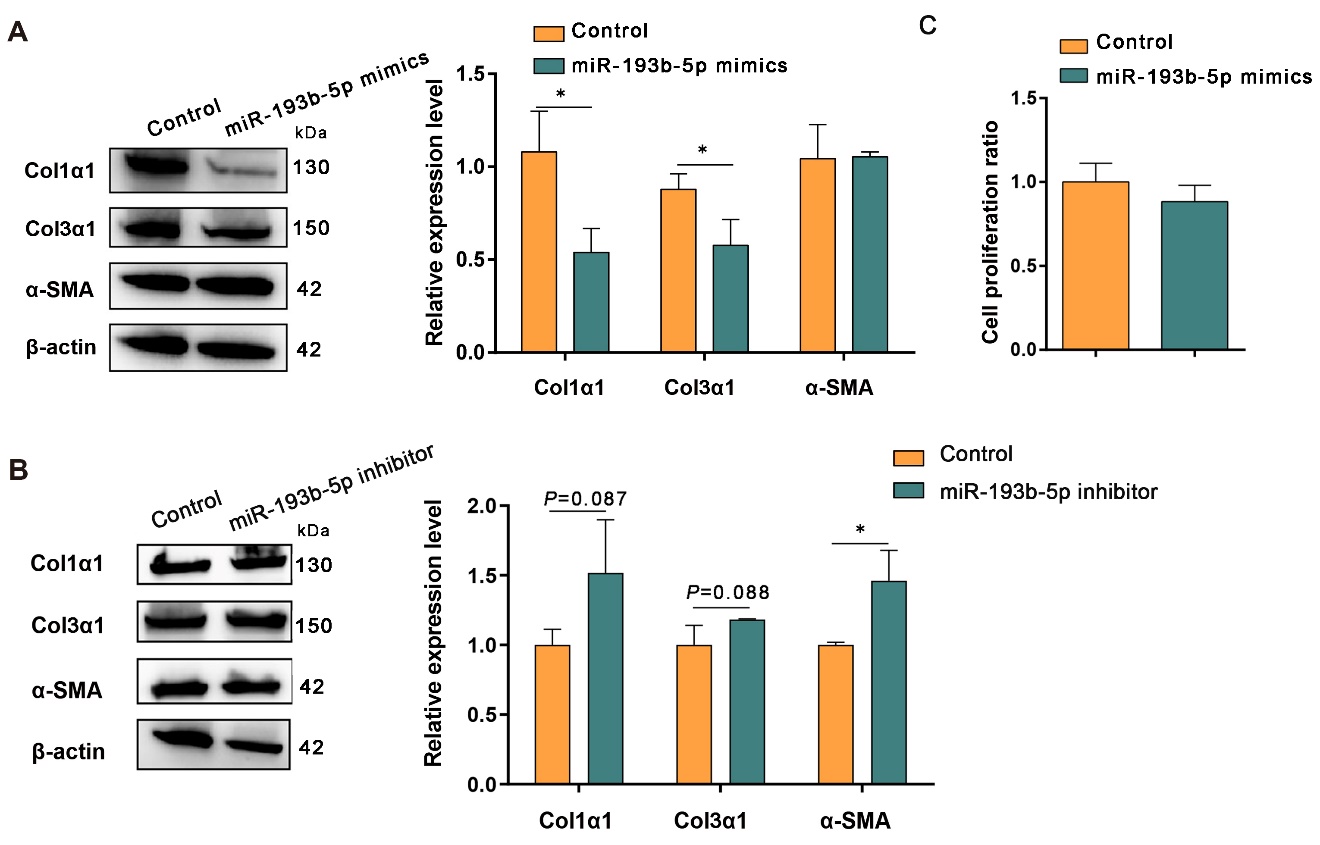
**

**Fig. S5 The effect of miR-193b-5p on ECM production and HSC proliferation** The expression levels of Col1α1, Col3α1, and α-SMA were comparatively assessed in HSCs after 24 h transfection with miR-193b-5p mimics and mimics negative control (control, **A**) or by miR-193b-5p inhibitor and inhibitor negative control (control, **B**) by Western blotting. CCK8 was used to detect HSC cell proliferation after transfection with miR-193b-5p mimics and mimics negative control (control, **C**). ‘*’, *P* < 0.05.

**Table S1 The shRNA sequences against gm40262**

| RNA | Sequence (5’-3’) |
| --- | --- |
| shRNA1 | GGAGTGCATTACCAGACAATTCAAGAGATTGTCTGGTAATGCACTCCTTTTTT |
| ShRNA2 | GCAATGGCCCTTGCTTAATAATTCAAGAGATTATTAAGCAAGGGCCATTGCTTTTTT |
| ShRNA3 | GAGGTGGTTTCTGTCTTTAATTTCAAGAGAATTAAAGACAGAAACCACCTCTTTTTT |

**Table S2 The siRNA sequences against gm40262**

| siRNA | Sense (5’- 3’) | Antisense (5’- 3’) |
| --- | --- | --- |
| si-gm40262-1 | GGAGUGCAUUACCAGACAATT | UUGUCUGGUAAUGCACUCCTT |
| si-gm40262-2 | CCACUACUAAACCUUCCAATT | UUGGAAGGUUUAGUAGUGGTT |
| si-gm40262-3 | GCUAUACUGAAGAGCAAUATT | UAUUGCUCUUCAGUAUAGCTT |
| si-gm40262-4 | GCAAUCUGCAUAGUGAAUATT | UAUUCACUAUGCAGAUUGCTT |

**Table S3 The primers used for qPCR**

| Target | Forward primer (5’-3’) | Reverse primer (5’-3’) |
| --- | --- | --- |
| Gm40262 | CATCTCAATCCTCCAGACCTC | GTCAGCGAACATTCTACCG |
| Collα1 | GCACGAGTCA CACCGGAAC | CCAATGTCCAAGGGAGCCAC |
| α-SMA | GTCCCAGACATCAGGGAGTAA | TCGGATACTTCAGCGTCAGGA |
| Col3α1 | TGGTCCTCAGGGTGTAAAGG | GTCCAGCATCACCTTTTGGT |
| TLR4 | TTCACCTCTGCCTTCACTA | GACACTACCACAATAACCTTCC |
| IL-1β | AATGCCACCTTTTGACAGTGATG | AGCTTCTCCACAGCCACAAT |
| TGF-β | CTTCAATACGTCAGACATTCGGG | GTAACGCCAGGAATTGTTGCTA |
| GAPDH | ACCACAGTCCATGCCATCAC | TCCACCACCCTGTTGCTGTA |

**Table S4 The probes used for fluorescence *in situ* hybridization**

| Probe | Sequences (5’-3’) |
| --- | --- |
| P1 | TGCTTGAAAAGGAGGGCATCTTAGTC |
|  | TCCCCCAGTGGACAGGTATTTGGTTA |
|  | GAACTGTTTGGGGTTTGGAAGGTTTAG |
| P2 | GTTTGTAAGTGTTTGTAAGTGTTTGTAAG |
| P3 | TTATGATGATGTATGATGATGT |

**Table S5 Potential inflammation-related targets of miR-193b-5p**

| Genes | Gene full name | Functions | Ref |
| --- | --- | --- | --- |
| STST3 | Signal transducers and activators of transcription 3 | Stimulate expression of innate immune mediators in liver | (1) |
| TLR4 | Toll-like receptor 4 | Core mediator of adaptive and innate immune reactions to LPS | (2, 3) |
| TGF-β | Transforming growth factor-beta | Regulate T-cells and dendritic cells, a key player in driving the adaptive immune response | (4) |
| SENP2 | Small ubiquitin-like modifier (SUMO)-specific Protease 2 | Play a critical role in antiviral innate immunity by desumoylating RIG-I and MDA5 | (5) |
| CTNNBIP1 | β-catenin interacting protein 1 | Knockout of CTNNBIP1 in adipose tissue alleviates fibro-inflammation in obese mice | (6) |
| STIM2 | Stromal interaction molecule 2 | Promote proinflammatory function | (7) |
| P2RX1 | Purinergic receptor P2X 1 | Inflammatory activation of immune cells | (8) |
| NTSR1 | Neurotensin high affinity receptor 1 | NTSR1 is expressed in inflammatory demyelinating lesions | (9) |
| NOS1 | Nitric oxide synthase 1 | NOS1 mediated regulation of AP1 that turns on the downstream cascade to generate the appropriate inflammatory responses | (10) |
| MYLK2 | Myosin light chain kinase 2 | Involved in the inflammatory pathway mediated by chemokine and cytokine signaling | (11) |
| Htr2b | 5-hydroxytryptamine (serotonin) receptor 2b | Promote M1 microglia polarization and neuroinflammation | (12) |
| Htr2a | 5-hydroxytryptamine (serotonin) receptor 2a | Restrict the JAK/STAT signaling cascade downstream of the IL-7/IL-7R pathway | (13) |
| GNAS | G-protein alpha-subunit | Promote STAT3 activation | (14) |
| Chrna7 | Cholinergic receptor nicotinic alpha 7 subunit | Involved in cholinergic anti-inflammatory pathway | (15) |
| CaMK4 | Calcium/calmodulin-dependent protein kinase IV | Promote the expansion of Th17 cells through the AKT/mTOR signaling pathway | (16) |
| TRAF6 | Tumor necrosis factor receptor-associated factor 6 | Mediate NF-κB activation | (17) |
| TGFBR2 | Type II serine/threonine kinase receptor | Activate the TGF-β signaling pathway | (18) |
| TCF7 | Transcription factor 7 | Regulate the expression of inflammatory factors | (19) |
| SUFU | Suppressor of fused | A key negative regulator of the Hh signaling pathway;  mediate EMT and Wnt/β-catenin activation in Hh-independent manner | (20, 21) |
| SMAD3 | SMAD family member 3 | Involved in the TGF-β signaling pathway | (22) |
| SMAD4 | SMAD family member 4 | Involved in the TGF-β signaling pathway | (22) |
| PTEN | Phosphatase and tensin homolog deleted on chromosome 10 | Regulate the PI3K/Akt prosurvival signaling | (23) |
| MAPK8 | Mitogen-activated protein kinase 8 | Down-regulation of MAPK8 inhibits inflammatory responses | (24) |
| CAMK2A | Calcium/calmodulin-dependent protein kinase II α | CAMK2A knockdown inhibits inflammation | (25) |
| HGF | Hepatocyte growth factor | Reduce accumulation of inflammation | (26) |
| MAP2K1 | Mitogen-activated protein kinases MEK1 | MAP2K1 deletion leads to prolonged MAP2K2-ERK1/2 activation, resulting in sustained inflammatory responses | (27) |
| MAPK3 | Mitogen-activated protein kinase 3 | Activate inflammatory responses | (28) |
| MMP9 | Matrix-metalloproteinase 9 | Regulates cellular responses to inflammation | (29) |
| PDCD4 | Programmed cell death 4 | PDCD4 siRNA inhibits LPS-induced increase of IL-1β, TNF-α production and p-NF-κB (p65) expression | (30) |
| PIK3CA | Phosphatidylinositol-4,5-bisphosphate 3-kinase catalytic subunit alpha | Activate the PIK3CA/Akt/NF-κB signaling | (31) |
| PRKCA | Protein kinase C alpha | PKC-α inhibitor attenuates CBNPs-induced inflammation by down-regulation of ROS | (32) |
| PRKCG | Protein kinase C gamma | Control the balance between pro-inflammatory and Treg cell subsets | (33) |
| THBS1 | Thrombospondin 1 | Enhance systemic inflammation | (34) |
| TIMP3 | Tissue inhibitor of metalloproteinase 3 | Control TNF levels *in vivo* | (35) |
| Wnt7a | Wnt family member 7A | Promote inflammation | (36) |
| APPL1 | Adaptor protein, phosphotyrosine interaction, PH domain and leucine zipper containing 1 | Prevent inflammation by dampening NFκB activation | (37) |
| BCR | BCR activator of RhoGEF and GTPase | Activate the NF-κB pathway | (38) |
| CDK6 | Cyclin-dependent kinase 6 | CDK6 inhibition attenuates IL-1β-induced inflammation | (39) |
| CEBPA | CCAAT enhancer binding protein alpha | Anti-inflammatory effects | (40) |

**References**

1. Hillmer EJ, Zhang H, Li HS, Watowich SS. 2016. STAT3 signaling in immunity. Cytokine Growth Factor Rev 31:1-15. https://doi.org/10.1016/j.cytogfr.2016.05.001.
2. Kuzmich NN, Sivak KV, Chubarev VN, Porozov YB, Savateeva-Lyubimova TN, Peri F. 2017. TLR4 Signaling Pathway Modulators as Potential Therapeutics in Inflammation and Sepsis. Vaccines (Basel) 5:34. https://doi.org/10.3390/vaccines5040034.
3. Khan HU, Aamir K, Jusuf PR, Sethi G, Sisinthy SP, Ghildyal R, Arya A.2021. Lauric acid ameliorates lipopolysaccharide (LPS)-induced liver inflammation by mediating TLR4/MyD88 pathway in Sprague Dawley (SD) rats. Life Sci 265:118750. https://doi.org/10.1016/j.lfs.2020.118750.
4. Worthington JJ, Fenton TM, Czajkowska BI, Klementowicz JE, Travis MA. 2012. Regulation of TGFβ in the immune system: an emerging role for integrins and dendritic cells. Immunobiology 217:1259-65. https://doi.org/10.1016/j.imbio.2012.06.009.
5. Wang K, Jiang Z, Lu X, Zhang Y, Yuan X, Luo D, Lin Z, Zuo Y, Luo Q. 2020. Cardiomyocyte-specific deletion of Senp2 contributes to CVB3 viral replication and inflammation. Int Immunopharmacol 88:106941. https://doi.org/10.1016/j.intimp.2020.106941.
6. Song Z, Liu N, He Y, Chen J, Li J, Wang F, Wu Z. 2023. Knockout of ICAT in Adipose Tissue Alleviates Fibro-inflammation in Obese Mice. Inflammation 46:404-417. https://doi.org/10.1007/s10753-022-01742-w.
7. Saint Fleur-Lominy S, Maus M, Vaeth M, Lange I, Zee I, Suh D, Liu C, Wu X, Tikhonova A, Aifantis I, Feske S. 2018. STIM1 and STIM2 Mediate Cancer-Induced Inflammation in T Cell Acute Lymphoblastic Leukemia. Cell Rep 24:3045-3060.e5. https://doi.org/10.1016/j.celrep.2018.08.030.
8. Wang X, Yuan X, Su Y, Hu J, Ji Q, Fu S, Li R, Hu L, Dai C. 2021. Targeting Purinergic Receptor P2RX1 Modulates Intestinal Microbiota and Alleviates Inflammation in Colitis. Front Immunol 12:696766. https://doi.org/10.3389/fimmu.2021.696766.
9. Soltys J, Knight J, Scharf E, Pitt D, Mao-Draayer Y. 2014. IFN-β alters neurotrophic factor expression in T cells isolated from multiple sclerosis patients - implication of novel neurotensin/NTSR1 pathway in neuroprotection. Am J Transl Res 6:312-9. PMID: 24936223.
10. Srivastava M, Baig MS. 2018. NOS1 mediates AP1 nuclear translocation and inflammatory response. Biomed Pharmacother 102:839-847. https://doi.org/10.1016/j.biopha.2018.03.069.
11. Sudigyo D, Rahmawati G, Setiasari DW, Poluan RH, Sesotyosari SL, Wardana T, Herawati C, Heriyanto DS, Indrasari SR, Afiahayati -, Astuti I, Haryana SM. 2020. Transcriptome Profile of Next Generation Sequence Data Related to Inflammation on Nasopharyngeal Carcinoma Cases in Indonesia. Asian Pac J Cancer Prev 21:2763-2769. https://doi.org/10.31557/APJCP.2020.21.9.2763.
12. Chen W, Gao X, Yang W, Xiao X, Pan X, Li H. 2024. Htr2b Promotes M1 Microglia Polarization and Neuroinflammation after Spinal Cord Injury via Inhibition of Neuregulin-1/ErbB Signaling. Mol Neurobiol 61:1643-1654. https://doi.org/10.1007/s12035-023-03656-6.
13. Wang Z, Yan C, Du Q, Huang Y, Li X, Zeng D, Mao R, Gurram RK, Cheng S, Gu W, Zhu L, Fan W, Ma L, Ling Z, Qiu J, Li D, Liu E, Zhang Y, Fang Y, Zhu J, Sun B. 2023. HTR2A agonists play a therapeutic role by restricting ILC2 activation in papain-induced lung inflammation. Cell Mol Immunol 20:404-418. https://doi.org/10.1038/s41423-023-00982-6.
14. Ding H, Zhang X, Su Y, Jia C, Dai C. 2020. GNAS promotes inflammation-related hepatocellular carcinoma progression by promoting STAT3 activation. Cell Mol Biol Lett 25:8. https://doi.org/10.1186/s11658-020-00204-1.
15. Yang Y, Eguchi A, Wan X, Chang L, Wang X, Qu Y, Mori C, Hashimoto K. 2023. A role of gut-microbiota-brain axis via subdiaphragmatic vagus nerve in depression-like phenotypes in Chrna7 knock-out mice. Prog Neuropsychopharmacol Biol Psychiatry 120:110652. https://doi.org/10.1016/j.pnpbp.2022.110652.
16. Chang S, Yin T, He F, Ding J, Shang Y, Yang J. 2020. CaMK4 promotes abortion-related Th17 cell imbalance by activating AKT/mTOR signaling pathway. Am J Reprod Immunol 84:e13315. https://doi.org/10.1111/aji.13315.
17. Lv Y, Kim K, Sheng Y, Cho J, Qian Z, Zhao YY, Hu G, Pan D, Malik AB, Hu G. 2018. YAP Controls Endothelial Activation and Vascular Inflammation Through TRAF6. Circ Res 123:43-56. https://doi.org/10.1161/CIRCRESAHA.118.313143.
18. Massagué J. 1998. TGF-beta signal transduction. Annu Rev Biochem 67:753-91. https://doi.org/10.1146/annurev.biochem.67.1.753.
19. Yao T, Zhang L, Fu Y, Yao L, Zhou C, Chen G. 2021. Saikosaponin-d Alleviates Renal Inflammation and Cell Apoptosis in a Mouse Model of Sepsis via TCF7/FOSL1/Matrix Metalloproteinase 9 Inhibition. Mol Cell Biol 41:e0033221. https://doi.org/10.1128/MCB.00332-21.
20. Huang D, Wang Y, Tang J, Luo S. 2018. Molecular mechanisms of suppressor of fused in regulating the hedgehog signalling pathway. Oncol Lett 15:6077-6086. https://doi.org/10.3892/ol.2018.8142.
21. Peng Y, Zhang X, Lin H, Deng S, Qin Y, Yuan Y, Feng X, Wang J, Chen W, Hu F, Yan R, Zhao Y, Cheng Y, Wei Y, Fan X, Ashktorab H, Smoot D, Li S, Meltzer SJ, Jin Z. 2020. SUFU mediates EMT and Wnt/β-catenin signaling pathway activation promoted by miRNA-324-5p in human gastric cancer. Cell Cycle 19:2720-2733. https://doi.org/10.1080/15384101.2020.1826632.
22. Lan HY. 2011. Diverse roles of TGF-β/Smads in renal fibrosis and inflammation. Int J Biol Sci 7:1056-67. https://doi.org/10.7150/ijbs.7.1056.
23. Piguet AC, Dufour JF. 2011. PI(3)K/PTEN/AKT pathway. J Hepatol 54:1317-9. https://doi.org/10.1016/j.jhep.2010.12.013.
24. Gu M, Liu K, Xiong H, You Q. 2024. MiR-130a-3p inhibits endothelial inflammation by regulating the expression of MAPK8 in endothelial cells. Heliyon 10:e24541. https://doi.org/10.1016/j.heliyon.2024.e24541.
25. Jin S, Tian S, Ding H, Yu Z, Li M. 2022. SNHG5 knockdown alleviates neuropathic pain induced by chronic constriction injury via sponging miR‑142‑5p and regulating the expression of CAMK2A. Mol Med Rep 26:221. https://doi.org/10.3892/mmr.2022.12737.
26. Yan L, He X, Tang Y, Zhao X, Luo G, Wang X. 2021. HGF can reduce accumulation of inflammation and regulate glucose homeostasis in T2D mice. J Physiol Biochem 77:613-624. https://doi.org/10.1007/s13105-021-00828-7.
27. Long ME, Gong KQ, Eddy WE, Volk JS, Morrell ED, Mikacenic C, West TE, Skerrett SJ, Charron J, Liles WC, Manicone AM. 2019. MEK1 regulates pulmonary macrophage inflammatory responses and resolution of acute lung injury. JCI Insight 4(23):e132377. https://doi.org/10.1172/jci.insight.132377.
28. Di XP, Jin X, Ai JZ, Xiang LY, Gao XS, Xiao KW, Li H, Luo DY, Wang KJ. 2022. YAP/Smad3 promotes pathological extracellular matrix microenviroment-induced bladder smooth muscle proliferation in bladder fibrosis progression. MedComm (2020) 3:e169. https://doi.org/10.1002/mco2.169.
29. Wang X, Yu YY, Lieu S, Yang F, Lang J, Lu C, Werb Z, Hu D, Miclau T, Marcucio R, Colnot C. 2013. MMP9 regulates the cellular response to inflammation after skeletal injury. Bone 52:111-9.https://doi.org/10.1016/j.bone.2012.09.018.
30. Liu H, Sun J, Gao L, Fan L, Chen D, Wu L. 2021. MicroRNA‑421 attenuates macrophage‑mediated inflammation by inhibiting PDCD4 in vitro. Mol Med Rep 24:527. https://doi.org/10.3892/mmr.2021.12166.
31. He R, Tang GL, Niu L, Ge C, Zhang XQ, Ji XF, Fang H, Luo ZL, Chen M, Shang XF. 2020. Quietness Circ 0000962 promoted nerve cell inflammation through PIK3CA/Akt/NF-κB signaling by miR-302b-3p in spinal cord injury. Ann Palliat Med 9:190-198. https://doi.org/10.21037/apm.2020.02.13.
32. Hsu HT, Tseng YT, Wong WJ, Liu CM, Lo YC. 2018. Resveratrol prevents nanoparticles-induced inflammation and oxidative stress via downregulation of PKC-α and NADPH oxidase in lung epithelial A549 cells. BMC Complement Altern Med 18:211. https://doi.org/10.1186/s12906-018-2278-6.
33. Zanin-Zhorov A, Blazar BR. 2021. ROCK2, a critical regulator of immune modulation and fibrosis has emerged as a therapeutic target in chronic graft-versus-host disease. Clin Immunol 230:108823. https://doi.org/10.1016/j.clim.2021.108823.
34. Hassan HM, Liang X, Xin J, Lu Y, Cai Q, Shi D, Ren K, Li J, Chen Q, Li J, Li P, Guo B, Yang H, Luo J, Yao H, Zhou X, Hu W, Jiang J, Li J. 2024. Thrombospondin 1 enhances systemic inflammation and disease severity in acute-on-chronic liver failure. BMC Med 22:95. https://doi.org/10.1186/s12916-024-03318-x.
35. Black RA. 2004. TIMP3 checks inflammation. Nat Genet 36:934-5. https://doi.org/10.1038/ng0904-934.
36. Wang W, Yan X, Lin Y, Ge H, Tan Q. 2018. Wnt7a promotes wound healing by regulation of angiogenesis and inflammation: Issues on diabetes and obesity. J Dermatol Sci S0923-1811(18)30103-8. https://doi.org/10.1016/j.jdermsci.2018.02.007.
37. Jiang X, Zhou Y, Wu KK, Chen Z, Xu A, Cheng KK. 2017. APPL1 prevents pancreatic beta cell death and inflammation by dampening NFκB activation in a mouse model of type 1 diabetes. Diabetologia 60:464-474. https://doi.org/10.1007/s00125-016-4185-z.
38. Cai J, Zhao C, Du Y, Huang Y, Zhao Q. 2019. Amentoflavone ameliorates cold stress-induced inflammation in lung by suppression of C3/BCR/NF-κB pathways. BMC Immunol 20:49. https://doi.org/10.1186/s12865-019-0331-y.
39. Sun H, Huang Z, Wu P, Chang Z, Liao W, Zhang Z. 2018. CDK6 and miR-320c Co-Regulate Chondrocyte Catabolism Through NF-κB Signaling Pathways. Cell Physiol Biochem 51:909-923. https://doi.org/10.1159/000495392.
40. Zhou J, Li H, Xia X, Herrera A, Pollock N, Reebye V, Sodergren MH, Dorman S, Littman BH, Doogan D, Huang KW, Habib R, Blakey D, Habib NA, Rossi JJ. 2019. Anti-inflammatory Activity of MTL-CEBPA, a Small Activating RNA Drug, in LPS-Stimulated Monocytes and Humanized Mice. Mol Ther 27:999-1016. https://doi.org/10.1016/j.ymthe.2019.02.018.
